# Nutrients and bioactive compounds in polycystic ovary syndrome: updated insights into effects and underlying mechanisms

**DOI:** 10.3389/fnut.2026.1697275

**Published:** 2026-02-09

**Authors:** Yanli Zhang, Baichao Shi, Yuan Tian, Shujun Xu, Hui Chang

**Affiliations:** 1 Graduate School, Heilongjiang University of Chinese Medicine, Harbin, China; 2 The First Affiliated Hospital, Heilongjiang University of Chinese Medicine, Harbin, China

**Keywords:** polycystic ovary syndrome, nutrients, vitamins, minerals, bioactive compounds, metabolism

## Abstract

Polycystic ovary syndrome (PCOS) is one of the most prevalent endocrine disorders affecting women of reproductive age. Its complex and heterogeneous clinical presentation has led international evidence-based guidelines to prioritize management strategies aimed at symptom control rather than cure, highlighting the need for novel therapeutic approaches. Women with PCOS frequently exhibit deficiencies in various vitamins and minerals, which are closely associated with the syndrome’s characteristic insulin resistance (IR) and endocrine disturbances. Consequently, nutritional supplementation may provide significant adjunctive benefits to conventional therapies. Accumulating evidence indicates that specific vitamins (e.g., E, K, D, B-8, B-9, B-12), minerals (e.g., selenium, chromium, zinc, calcium, magnesium), and other bioactive compounds (e.g., melatonin, *ω*-3 polyunsaturated fatty acids, coenzyme Q10, N-acetylcysteine) can ameliorate core PCOS manifestations. Their potential mechanisms involve regulating glucose and lipid metabolism, correcting hormonal imbalances, attenuating oxidative stress and chronic inflammation, and beneficially modulating gut microbiota composition. This narrative review synthesizes current evidence on the roles of these nutrients in PCOS, elucidates their potential mechanistic pathways, and discusses their clinical applicability, thereby providing insights for integrative management and future research directions.

## 1 Introduction

Polycystic ovary syndrome (PCOS) is one of the most common endocrine disorders affecting women of reproductive age, characterized by a heterogeneous clinical presentation involving hormonal, reproductive, and metabolic dysregulation (1). Typical hormonal abnormalities include elevated fasting insulin (FINS), luteinizing hormone (LH), free testosterone (FT), and estrogen levels, often accompanied by suboptimal follicle-stimulating hormone (FSH) secretion and an elevated LH/FSH ratio (2). Clinical manifestations of this endocrine imbalance are diverse, with infertility, clinical hyperandrogenism (e.g., hirsutism, alopecia, and acne), and menstrual irregularities being highly prevalent among affected individuals (3). Beyond reproductive sequelae, PCOS is strongly associated with obesity, insulin resistance (IR), and disturbances in glucose and lipid metabolism (4). Moreover, elevated levels of pro-inflammatory mediators, obesity, and IR interact synergistically, exacerbating the underlying pathophysiology. Concurrently, increased oxidative stress (OS) and inflammation contribute to the deterioration of ovarian function (5). These intertwined endocrine and metabolic alterations collectively heighten the long-term risk of type 2 diabetes, metabolic syndrome (MetS), and cardiovascular disease in this population (6).

To date, PCOS management strategies are primarily tailored to individual pathophysiological, clinical, and metabolic profiles. Lifestyle modifications—including exercise and dietary interventions—constitute the first-line therapeutic approach, while pharmacological agents are used as adjunctive treatment. Although pharmacotherapy demonstrates efficacy, treatment discontinuation is often associated with symptom recurrence and potential adverse effects (7). In recent years, growing interest has emerged regarding the potential role of vitamin, mineral, and nutrient supplementation in PCOS management. While numerous meta-analyses support the efficacy of specific nutritional interventions in ameliorating PCOS symptomatology (8), a critical synthesis evaluating their overall clinical applicability remains lacking. Thus, this review aims to synthesize and critically evaluate recent evidence on the utility of nutritional supplementation in the treatment and management of PCOS, with the goal of informing the integration of evidence-based nutritional approaches into clinical practice and guidelines.

## 2 Methods

### 2.1 Literature search strategy

A systematic literature search was conducted using electronic databases including PubMed, Web of Science, and the China National Knowledge Infrastructure (CNKI) for articles published up to August 2025. The search combined keywords and Medical Subject Headings (MeSH) related to “Polycystic Ovary Syndrome” or “PCOS” with terms referring to individual nutrients and their mechanisms.

### 2.2 Study selection

The initial search prioritized meta-analyses, systematic reviews, and randomized controlled trials (RCTs) to ensure a high evidence level. Preclinical studies were selectively included to elucidate underlying molecular mechanisms when clinical data were limited or unavailable.

### 2.3 Inclusion and exclusion criteria

Studies were included if they investigated the effects or mechanisms of selected nutrients or compounds in PCOS patients or relevant PCOS models. Studies without accessible full text were excluded.

### 2.4 Data extraction and synthesis

As a narrative review, data pertaining to study design, population, intervention (dose and duration), key findings, and proposed mechanisms were extracted and synthesized thematically.

## 3 PCOS pathophysiology

PCOS is a chronic, heterogeneous disorder characterized by hyperandrogenism (HA) as a primary clinical feature. Elevated androgen levels are observed in approximately 75–90% of PCOS women with oligomenorrhea, often correlating positively with phenotypic severity (9). Dysregulation of the hypothalamic–pituitary-ovarian axis, driven by increased LH secretion, stimulates theca cell hyperplasia and enhances the expression of enzymes critical for androgen biosynthesis, thereby promoting excessive androgen production (10). Altered cortisol metabolism represents another mechanism contributing to androgen excess in PCOS. Enhanced cortisol inactivation via 5α-reductase attenuates negative feedback on adrenocorticotropic hormone secretion, maintaining normal plasma cortisol concentrations while increasing androgen synthesis (11).

IR constitutes a core pathophysiological feature in PCOS. Prevalent among affected women, IR selectively impairs insulin signaling in target tissues including adipose tissue, liver, skeletal muscle, ovaries, and the pituitary gland (12). This defect disrupts both glucose and lipid metabolism. A significant correlation exists between testosterone (T) and circulating insulin levels. Increased androgen production, along with consequent induction of abdominal adiposity, contributes to systemic IR in PCOS. Concurrently, hyperinsulinemia synergizes with LH at the ovarian level and suppresses hepatic sex hormone-binding globulin (SHBG) production, further exacerbating HA (13). Similarly, Obesity and HA, potentiated by IR, activate nuclear factor kappa B (NF-κB), triggering the release of pro-inflammatory mediators such as leptin, interleukin-6 (IL-6), and tumor necrosis factor alpha (TNF-*α*). This establishes a state of chronic low-grade inflammation and OS, which concurrently impairs insulin signaling and promotes free fatty acid release from adipose tissue. These processes further potentiate ectopic fat deposition and worsen IR, creating a self-reinforcing cycle (14, 15).

In summary, IR/hyperinsulinemia, hyperandrogenism, obesity, chronic inflammation, and OS engage in a complex, synergistic interplay. This interconnected pathophysiology drives the progression and clinical manifestations of PCOS.

## 4 Vitamins and PCOS

### 4.1 Vitamin E

Vitamin E (tocopherol), a fat-soluble vitamin, is primarily stored in the liver and gradually released into the bloodstream to maintain physiological concentrations. It exerts crucial antioxidant effects by neutralizing free radicals, inhibiting lipid peroxidation, and promoting cell renewal (16). Beyond supplementation, dietary intake represents a vital source of vitamin E. Primary food sources include vegetable oils (e.g., wheat germ oil, sunflower oil), nuts (almonds, walnuts), seeds (sunflower seeds), and dark green leafy vegetables (spinach) (17).

In the clinical management of PCOS, vitamin E has demonstrated multifaceted potential for improvement. A retrospective cohort study revealed that short-term vitamin E supplementation (100 mg/day, orally) in 321 infertile women with PCOS undergoing ovulation induction with clomiphene citrate and human menopausal gonadotropin (HMG) reduced the required HMG dosage without compromising reproductive outcomes, possibly by alleviating OS (18). In a related context, a 12-week intervention combining *ω*-3 polyunsaturated fatty acids (PUFAs) (1,000 mg/day) with vitamin E (400 IU) was reported to significantly improve lipid profiles—reducing serum triglycerides (TG), very-low-density lipoprotein cholesterol (VLDL-C), total cholesterol (TC), low-density lipoprotein cholesterol (LDL-C) and the TC to high-density lipoprotein cholesterol (HDL-C) ratio—and enhance total antioxidant capacity (TAC) and decreasing malondialdehyde (MDA) levels (19). This combination upregulated the expression of peroxisome proliferator-activated receptor gamma (PPAR-*γ*) and downregulated pro-inflammatory cytokines, including IL-8 and TNF-*α*, in peripheral blood mononuclear cells (PBMCs) of PCOS patients (20). A meta-analysis indicated that vitamin E supplementation effectively declined T, LH, LDL, and TG levels while increasing progesterone (P) and FSH concentrations in PCOS patients (21). Beneficial effects were also observed on waist circumference (WC), insulin levels and the homeostasis model assessment of insulin resistance (HOMA-IR) (21). Another meta-analysis further confirmed that both vitamin E monotherapy and its combination with *ω*-3 PUFAs or magnesium significantly lowered TG, LDL, TC, high-sensitivity C-reactive protein (hs-CRP) levels, and hirsutism scores, while markedly augmenting nitric oxide (NO) concentrations compared to placebo (22). The inconsistencies observed across these studies may be related to the duration. Collectively, the data suggest that vitamin E may alleviate IR and ameliorate endocrine imbalances in PCOS, potentially by activating antioxidant regulatory mechanisms, attenuating chronic inflammatory responses, and reducing lipid peroxidation and lipogenesis.

Notably, synergistic interactions between vitamin E and other nutrients may hold significant relevance. A classic synergistic relationship exists with vitamin C in antioxidant defense; vitamin C contributes to regenerating oxidized vitamin E, thereby restoring its activity and enhancing cellular membrane integrity protection (23). A close functional interaction also exists with selenium (24). As an essential component of glutathione peroxidase (GPX), adequate selenium status supports vitamin E in performing its core function of inhibiting lipid peroxidation more effectively (24–26). Furthermore, combined supplementation with vitamin E and *ω*-3 PUFAs may be superior to monotherapy, as vitamin E helps prevent the *in vivo* oxidation of ω-3 PUFAs, while the two nutrients together may produce a synergistic effect in mitigating inflammation and modulating lipid metabolism (22).

Despite these benefits, potential risks must be considered. A meta-analysis of 19 RCTs involving 135,967 participants examined the dose–response relationship between vitamin E supplementation and all-cause mortality. The analysis revealed that high-dose vitamin E (≥ 400 IU/day) was significantly associated with an increase in all-cause mortality, with a pooled risk difference of 39 additional deaths per 10,000 individuals. Dose–response analysis further indicated a statistically significant augmentation in mortality risk at doses above 150 IU/day (27). Therefore, for nutritional intervention in PCOS patients, safe and effective dosing should be determined under clinical or nutritional guidance, tailored to individual circumstances, with priority given to obtaining vitamin E through a balanced diet.

### 4.2 Vitamin K

Vitamin K exists primarily in two natural forms: vitamin K1 (phylloquinone) and vitamin K2 (menaquinones) (28). Vitamin K1, predominantly found in green leafy vegetables, serves as the primary dietary source of this vitamin and is also synthesized in algae through photosynthesis. In contrast, Vitamin K2 (VK2) is mainly produced by the human gut microbiota. Dietary VK2 is derived primarily from microbial sources and is commonly present in fermented foods—such as cheese, curds, cream, sour cream, butter, and natto (a fermented soybean product)—as well as in animal-based products including eggs, chicken, ham, and liver (28, 29).

Renowned for its classic role as a cofactor for *γ*-glutamyl carboxylase in maintaining normal coagulation (30, 31), vitamin K is also implicated in regulating vascular and bone calcification. It serves as the essential cofactor for the carboxylation of proteins like osteocalcin—the most abundant non-collagenous protein in bone (30). The carboxylated form of osteocalcin is associated not only with bone mineralization but also with promoting *β*-cell proliferation and insulin secretion, thereby influencing metabolism and reproductive function (32).

In clinical intervention studies for PCOS, vitamin K has shown potential for improving metabolic and endocrine parameters. A RCT demonstrated that daily supplementation with 90 μg of menaquinone-7 (MK-7, a form of vitamin K2) for 8 weeks significantly dropped FINS, HOMA-IR, homeostasis model of assessment *β*-cell function (HOMA-β), TG, dihydrotestosterone (DHT), free androgen index (FAI), and body fat mass, while boosting the quantitative insulin sensitivity check index (QUICKI), sex hormone-binding globulin (SHBG) activity and skeletal muscle in women with PCOS (33). These findings imply beneficial effects on body composition, insulin sensitivity, and hyperandrogenemia in PCOS patients.

The beneficial effects of vitamin K are closely associated with its modulation of the gut microbiota. Proposed mechanisms include: firstly, vitamin K exerts the inhibition of lipopolysaccharide (LPS)-induced nucleotide-binding oligomerization domain-like receptor protein 3 (NLRP3) inflammasome activation and the suppression of NF-κB pathway signaling, thereby decreasing pro-inflammatory cytokines such as IL-6 and TNF-*α* (34). Secondly, in terms of antioxidant activity, vitamin K enhances the activities of superoxide dismutase (SOD), glutathione (GSH), GPX, and catalase (CAT), while simultaneously suppressing the expression of inducible nitric oxide synthase (iNOS), cyclooxygenase-2 (COX-2), p38 mitogen-activated protein kinase (MAPK), reactive oxygen species (ROS), and caspase-1, thus mitigating OS damage (34). Moreover, vitamin K raises the abundance of beneficial gut bacteria (such as *Ruminococcaceae* and *Lactobacillaceae*), depresses the population of potential pathogens (e.g., *Desulfovibrio* and *Escherichia coli*), and promotes the secretion of butyrate among short-chain fatty acids (SCFAs), collectively helping to restore intestinal barrier integrity and alleviate systemic inflammation (34, 35).

In terms of nutrient interactions, vitamin D and vitamin K exhibit a well-recognized synergy in bone and cardiovascular health. Vitamin D stimulates the synthesis of osteocalcin, while vitamin K is responsible for activating (carboxylating) this protein, facilitating the proper deposition of calcium into bone (36). Magnesium may indirectly support the function of vitamin K-dependent proteins as a cofactor for relevant metabolic enzymes.

Vitamin K displays a notably low systemic toxicity profile, with no adverse effects reported in animals even at high doses (2 g/kg body weight) over 1 month (37), although exceptionally high doses may induce hemorrhage and anemia (38). Human data align with this favorable safety profile; for instance, daily supplementation with 10 mg of vitamin K1 for 1 month produced no adverse effects (39). Intravenous vitamin K1 preparations, which are often aqueous colloidal suspensions, can occasionally cause anaphylactoid reactions, a risk attributed to their formulation. Overall, there are no widespread reports of toxicity from dietary intake or conventional supplementation of vitamin K in healthy populations, indicating a relatively wide safety margin.

The anti-inflammatory, antioxidant, and gut microbiota-modulating properties of vitamin K collectively propose a potential therapeutic role in PCOS. Nonetheless, the current body of evidence, particularly from high-quality clinical studies, is insufficient to draw definitive conclusions. Therefore, well-designed, large-scale randomized controlled trials are warranted to validate its efficacy in improving PCOS-related clinical outcomes and to further elucidate its underlying molecular and physiological mechanisms.

### 4.3 Vitamin D

Vitamin D is obtained through two primary routes: endogenous synthesis and dietary intake. The predominant source is endogenous production, which occurs when the skin is exposed to ultraviolet B (UVB) radiation from sunlight (40). In contrast, dietary sources are relatively limited; they primarily include oily fish, animal liver, egg yolks, and vitamin D-fortified products such as dairy and formulated foods.

Substantial evidence indicates a high prevalence of vitamin D deficiency in women with PCOS, closely associated with clinical manifestations and metabolic disturbances (32). A retrospective study showed that serum 25-hydroxyvitamin D3 [25-(OH) D3] concentrations were lower in PCOS patients than in healthy women, and the pregnancy rate was significantly declined in the vitamin D-deficient group (41). Supplementation with 4,000 IU/day of vitamin D for 2 months significantly increased serum 25-(OH) D3 levels and markedly decreased the LH/FSH ratio, LH, and T concentrations, and improved pregnancy rates following ovulation induction (41). Another clinical study reported that daily supplementation with 2000 IU of vitamin D for 12 weeks resulted in significant reductions in body mass index (BMI), waist-to-hip ratio, FINS, HOMA-IR, TG, TC, and LDL-C in women with PCOS (42). A meta-analysis of multiple RCTs confirmed that vitamin D supplementation significantly increased endometrial thickness and effectively diminished levels of hs-CRP, parathyroid hormone (PTH), TC, and total testosterone (TT) in PCOS patients (43). Common interventional regimens include vitamin D supplementation daily 4,000 IU or weekly 50,000 IU for 2 to 6 months, frequently combined with pharmaceutical agents such as ethinylestradiol/cyproterone acetate and clomiphene, or metformin along with calcium supplements, to synergistically improve endocrine and metabolic parameters. This integrated approach revealed positive effects on follicular development maturation and the regularization of menstrual cycles (44, 45).

The mechanisms through which vitamin D ameliorates the pathological state of PCOS are complex and multifaceted. In glucose metabolism, vitamin D response elements in the insulin gene promoter allow supplementation to directly stimulate insulin secretion, upregulate *β*-cell proliferation genes, and boost peripheral insulin sensitivity (46). Regarding its anti-inflammatory and anti-androgenic effects, vitamin D potentially diminishes advanced glycation end products (AGEs)-induced androgen synthesis via downregulating the expression of the receptor for advanced glycation end products (RAGE) and further lowers pro-inflammatory cytokine levels via inhibiting the activity of the phosphatidylinositol-3-kinase (PI3K)/protein kinase B (AKT) signaling pathway, consequently improving glucose homeostasis (47). Concerning mitochondrial function and ovarian health, vitamin D3 can activate the MAPK signaling pathway in granulosa cells from a dehydroepiandrosterone (DHEA)-induced PCOS mouse model, promoting mitochondrial biogenesis, upregulating key factors such as peroxisome proliferator-activated receptor gamma coactivator 1-alpha (PGC-1α) and nuclear respiratory factor 1 (NRF-1), reducing ROS levels, restoring mitochondrial function, and decreasing the apoptosis rate of granulosa cells (48).

The biological functions of vitamin D rely on synergistic interactions with other nutrients. The classic synergy with calcium involves vitamin D enhancing intestinal calcium absorption, while adequate calcium levels are fundamental for vitamin D’s skeletal benefits. Additionally, magnesium, serving as a cofactor for numerous enzymes, participates in the activation of vitamin D within the body, and its deficiency may consequently impair vitamin D functionality (49).

Despite the promising potential of vitamin D, potential risks associated with its supplementation warrant consideration. Prolonged, excessively high-dose supplementation (daily intake exceeding 50,000 IU) may lead to toxicity, causing hypercalcemia, hypercalciuria, and associated symptoms such as nausea, vomiting, kidney stones, and ectopic calcification of soft tissues (50). Personalized supplementation guided by monitoring serum 25-(OH) D3 levels is strongly recommended.

While mechanistic insights are promising, translating these findings into definitive clinical practice for PCOS has yet to be conclusively established. Robust evidence from large-scale, rigorous RCTs is still needed to confirm its therapeutic value, particularly regarding optimal dosing, the superiority of single-agent versus combination therapy, and the persistence of long-term benefits.

### 4.4 Inositol (vitamin B-8)

Inositol, a naturally occurring sugar alcohol, is widely distributed in various foods, with rich dietary sources including cereals, legumes, nuts, citrus fruits, and animal organs (51). Of the nine stereoisomers of inositol, myo-inositol (MI) and D-chiro-inositol (DCI) are the most abundant and physiologically significant. Maintaining a specific physiological ratio between MI and DCI is crucial for metabolic and reproductive health. In the ovaries of healthy women, the MI to DCI ratio is approximately 100:1; however, this ratio is significantly disrupted in women with PCOS, potentially dropping to as low as 0.2:1 (52), hinting that hyperinsulinemia in PCOS may pathologically heighten the conversion of MI to DCI, contributing to impaired glucose metabolism and reproductive dysfunction.

Clinical studies have confirmed the beneficial effects of inositol supplementation on improving PCOS symptoms. An RCT involving 34 PCOS patients aged 20–40 years showed that daily administration of MI + DCI (2,200 mg + 55 mg, 40:1 ratio) along with folic acid (400 μg) for 3 months significantly decreased BMI, HOMA-IR, insulin, TT, FT, and LH, while elevating levels of SHBG and estradiol (53). Beyond these parameters, a meta-analysis further indicated diminutions in androstenedione, blood glucose levels, and the area under the curve for insulin (54). A 6-month randomized study in 70 young PCOS women aged 15–24 with menstrual irregularities compared MI + DCI (550 mg + 150 mg, ratio 3.6:1) twice daily versus a combined hormonal contraceptive (CHC; containing 20 μg ethinylestradiol and 3 mg drospirenone) once daily; MI + DCI effectively restored regular menstrual cycles and significantly lowered serum anti-Müllerian hormone (AMH) levels and HOMA-IR (55). A meta-analysis revealed that MI/DCI supplementation significantly improved clinical pregnancy rates and high-quality embryo rates in women with PCOS, and reduced antral follicles and AMH levels (56).

Mechanistically, MI and DCI exhibit distinct yet complementary physiological roles. MI primarily functions as a “glucose sensor,” boosting cellular glucose uptake by promoting the translocation of glucose transporter type 4 (GLUT4) to the cell membrane. It also serves as a precursor for inositol phosphoglycan (IPG) mediators, restoring deficient ovarian synthesis of inositol trisphosphate (IP₃) and diacylglycerol (DAG), thereby regulating calcium release and protein kinase C (PKC) activation. This enhances FSH signal transduction, stimulates aromatase, curtails androgen production, and improves folliculogenesis and oocyte maturation (57, 58). DCI acts mainly as a “glycogen synthesizer,” responsible for mediating insulin-stimulated glycogen synthesis. However, elevated DCI levels in the ovary may inhibit aromatase activity and impede the conversion of androgens to estrogens, directly stimulating androgen synthesis, contributing to HA (57). Thus, supplementation with MI alone or in combination with DCI at a physiological ratio can help correct the underlying IR and hyperandrogenic state in PCOS.

PCOS patients frequently present with a state of OS and chronic low-grade inflammation. Studies indicate that DCI may intensify TAC by elevating the activities of key antioxidant enzymes such as SOD and CAT, while curbing ROS and MDA, potentially via the forkhead box O (FOXO)/DAF-16 and nuclear factor erythroid 2-related factor 2 (Nrf2)/SKN-1 signaling pathways (59). In PCOS model oocytes, MI (0.36 mg/g) significantly promoted oocyte maturation, increased adenosine triphosphate (ATP) and GSH content, and concurrently decreased ROS accumulation (60). In a letrozole-induced PCOS rat model, DCI (50 mg/kg) alone or in combination with Ecklonia cava extract (250 mg/kg) significantly reduced pro-inflammatory cytokine TNF-*α* and IL-6 levels, attenuating systemic inflammation (61).

The synergistic effect between inositol and folic acid has garnered significant attention. The combination of inositol and folic acid may exhibit complementary actions in collectively diminishing homocysteine (Hcy) levels, improving oocyte quality, and enhancing ovulatory function (60), leading to superior clinical outcomes.

Regarding safety and potential risks, inositol is generally well-tolerated. Adverse effects are primarily mild gastrointestinal symptoms—such as nausea, flatulence, and diarrhea—which have been reported only at high doses (12 g/day), without dose-dependent severity increase (62). Nevertheless, the long-term safety profile and the optimal upper dosage limit for prolonged supplementation remain unclear. Therefore, further large-scale, high-quality RCTs are warranted to systematically evaluate the long-term safety of inositol supplementation and to precisely determine the optimal dosages and time-dependent effects for improving metabolic and hormonal parameters in PCOS patients.

### 4.5 Folic acid (vitamin B-9)

Folic acid (vitamin B9) is a water-soluble vitamin, is found in dark green leafy vegetables, legumes, and animal liver/kidney (63). Naturally occurring folate in food exists in reduced forms, whereas the more stable synthetic form—folic acid (pteroylglutamic acid)—is typically used in dietary supplements and fortified foods. Folic acid exerts various physiological effects in the body, including anti-inflammatory and antioxidant activities, as well as cardiovascular protective effects, underscoring its potential applicability in ameliorating PCOS-related pathological conditions (64).

Clinical evidence supports the beneficial role of folic acid supplementation in PCOS management. A study in 150 obese PCOS patients demonstrated that daily supplementation with 1 mg or 5 mg of folic acid for 2 months significantly lowered serum levels of hs-CRP, IL-18, and TNF-*α* and insulin, while improving the HOMA-IR. Concurrently, the reductions in TC and LDL levels were more pronounced in the high-dose group compared to the low-dose group (65). In parallel, a study in PCOS women with hyperhomocysteinemia found that 1 mg folic acid daily for 3 months effectively decreased Hcy levels in all participants, with a more significant diminution observed in non-IR women, implying that folic acid supplementation contributes to improving the body’s methylation status and mitigates associated vascular endothelial damage (66). These findings manifest that folic acid confers combined anti-inflammatory, metabolic, and homocysteine-lowering benefits in women with PCOS.

The potential mechanisms by which folic acid ameliorates PCOS involve multiple signaling pathways. In terms of anti-inflammatory effects, folic acid suppresses the activation of the NF-κB pathway induced by ROS and Hcy, thereby downregulating the expression and secretion of pro-inflammatory cytokines such as TNF-*α*, IL-6, and IL-1β (67). Regarding the regulation of lipid metabolism and energy homeostasis, folic acid promotes the phosphorylation of AMP-activated protein kinase (AMPK) and its downstream target acetyl-CoA carboxylase (ACC), while increasing the expression of carnitine palmitoyltransferase 1 (CPT1), consequently enhancing fatty acid oxidation and improving lipid metabolism (68). In addition, to strengthen antioxidant defense, folic acid upregulates the expression of Nrf2 and its downstream effectors heme oxygenase-1 (HO-1) and NAD(P)H quinone dehydrogenase 1 (NQO1), while reducing OS markers such as MDA levels, thereby attenuating high-fat diet-induced OS and chronic inflammation (68).

Folic acid exhibits important synergistic interactions with other nutrients, among which its interplay with vitamin B12 is particularly critical. Vitamin B12 is an essential cofactor in the folic acid metabolic cycle, both involved in the remethylation of Hcy to methionine (69). Vitamin B12 deficiency can cause a “methylfolate trap,” a condition where folic acid becomes metabolically trapped and functionally impaired, making it difficult to effectively lower Hcy levels even with folic acid supplementation (70). In the clinical management of PCOS, combined assessment may yield greater benefits than folic acid supplementation alone.

Regarding potential risks and precautions, folic acid supplementation at conventional doses (e.g., 0.4–1 mg daily) is considered safe for the general population. However, long-term use of very high doses (exceeding 5 mg per day) may pose certain risks, such as masking the early hematological signs of vitamin B12 deficiency, thereby potentially delaying the diagnosis of its neurological complications (69). For PCOS patients, it is advisable to determine the appropriate supplementation dosage under medical supervision, taking into account individual factors such as pregnancy planning status and the presence of MTHFR gene mutations.

In brief, folic acid may play a beneficial role in ameliorating core pathological features of PCOS, including IR and chronic low-grade inflammation, likely mediated via its anti-inflammatory, antioxidant, and lipid metabolism-regulating properties.

### 4.6 Vitamin B-12

Vitamin B12 (cobalamin), a water-soluble vitamin, is almost exclusively derived from animal-based foods such as meat, poultry, fish, eggs, and dairy products (71). Consequently, strict vegetarians represent a high-risk population for deficiency (72). Physiologically, it serves as an essential cofactor in the conversion of methylmalonyl-CoA to succinyl-CoA—a substrate for the tricarboxylic acid cycle—and plays fundamental roles in DNA and protein synthesis, methylation cycles, and folic acid metabolism (73).

An observational study revealed that over 60% of women with PCOS had low vitamin B12 levels (300 pg./mL) accompanied by elevated Hcy. The study also reported a positive correlation between vitamin B12 and LDL-c, whereas inverse associations with AMH and 17-hydroxyprogesterone (17-OHP) levels (74). A study focusing on vegetarian PCOS patients not only confirmed prior findings of disturbed Hcy and methylmalonic acid (MMA) metabolism but also documented reduced circulating vitamin B12 and holo-transcobalamin (HTC). This compromised vitamin B12 status was associated with increased HOMA-IR and heightened levels of pro-inflammatory markers, including monocyte chemoattractant protein-1 (MCP-1), hs-CRP, TNF-*α*, and IL-6 (72).

Supplementation with vitamin B12 demonstrates multi-faceted potential in ameliorating PCOS manifestations. Metabolically, inadequate vitamin B12 status is associated with the accumulation of MMA, a specific functional marker of vitamin B12 deficiency (75). MMA acts as an inhibitor of CPT1 (a rate-limiting enzyme), impairing fatty acid *β*-oxidation, and potentially exacerbating lipid accumulation in the liver and peripheral tissues, thereby aggravating IR (75, 76). Endocrinologically, supplementation contributes to the reduction of AMH and 17-OHP levels, thereby correcting sex hormone imbalances (74). Regarding its anti-inflammatory properties, supplementation effectively lowers levels of inflammatory mediators including hs-CRP, TNF-*α*, and IL-6 (72). Vitamin B12 also modulates gut microbiota, such as increasing the Firmicutes-to-Bacteroidetes ratio, elevating the relative abundance of *Faecalibacterium*, decreasing *Bacteroides* populations, and promoting the production of SCFAs, particularly butyrate (77). These microbial shifts are implicated in the regulation of energy metabolism in PCOS patients.

The interaction between vitamin B12 and folic acid represents one of their core biological relationships. Both nutrients work in close coordination within the “methylation cycle,” where vitamin B12 acts as an essential coenzyme for methionine synthase. Facilitates the transfer of a methyl group from 5-methyltetrahydrofolate (5-methyl-THF) to homocysteine, forming methionine. Concurrently, vitamin B12 is utilized in the mitochondrial conversion (73). Thus, vitamin B12 deficiency can impair folic acid functionality. In the management of PCOS, combined supplementation with vitamin B12 and folic acid is generally more effective than monotherapy in correcting hyperhomocysteinemia.

Vitamin B12 has a high safety profile with no established observable adverse effect level. Routine oral supplementation (35 μg) is generally considered safe even at relatively high doses (1,000 μg) (78). However, severe vitamin B12 deficiency, daily oral supplementation with 500–2,000 μg is often recommended and demonstrates comparable efficacy to sublingual or intramuscular administration (78). Prior to initiating long-term, high-dose supplementation, it is advisable to assess serum vitamin B12 levels to confirm deficiency and guide personalized dosing.

Overall, vitamin B12 may alleviate common clinical manifestations of PCOS through multiple mechanisms, including the regulation of energy metabolism, chronic inflammation, endocrine balance, and gut microbiota composition. Future well-designed RCTs are still required to clarify its optimal supplementation strategy and long-term therapeutic efficacy.

## 5 Minerals and PCOS

### 5.1 Selenium

Selenium is an essential trace element for human health, primarily obtained from dietary sources such as Brazil nuts, cereals, meat, fish, and eggs (79). As a key component of antioxidant enzymes like GPX, selenium plays a vital role in cellular redox homeostasis. Clinical observational studies have elucidated that plasma selenium concentrations are significantly reduced in women with PCOS compared to age- and BMI-matched healthy controls (80). Moreover, selenium levels display a negative correlation with both LH and TT (80). Another investigation revealed that PCOS women with MetS had significantly lower dietary selenium intake compared to their non-MetS PCOS counterparts (81), suggesting a potential link between selenium deficiency and PCOS pathophysiology.

Supplementation with selenium has demonstrated beneficial effects in the clinical management of PCOS. Systematic reviews and meta-analyses of studies involving PCOS patients have consistently shown that selenium supplementation significantly enhances TAC while effectively decreasing serum levels of TT and cholesterol (82, 83). In intervention studies, selenium is commonly administered as selenomethionine or sodium selenite 200 μg/day for 8 weeks has been associated with improvements in metabolic parameters (84). Additionally, a clinical trial found that PCOS patients receiving selenium-enriched yeast tablets (60 μg/ day) for 12 weeks exhibited significant diminutions in asymmetric dimethylarginine (ADMA), TT, and the apolipoprotein B (ApoB)/apolipoprotein A1 (ApoA1) ratio (85).

The underlying mechanisms of selenium involve multiple pathways. In glucose metabolism, selenium upregulates the gene expression levels of PPAR-*γ* and GLUT1, thereby facilitating cellular glucose uptake and ameliorating glucose metabolic disorders (86). Regarding lipid and energy metabolism, selenium/selenoprotein P (Sepp1) effectively lessens lipid profiles through downregulating key molecules such as the LDL receptors and inhibiting the phosphorylation of critical energy metabolism regulators, including AKT and AMPK (86, 87). Additionally, Selenium intake boosts the expression and activity of GPX1. Through its antioxidant properties, GPX1 diminishes intracellular H₂O₂ generation, thereby suppressing pancreatic islet inflammation and OS, which collectively contribute to the protection of pancreatic *β*-cells (79).

Selenium exhibits significant biological interactions with other essential nutrients. It functions synergistically with iodine in the synthesis and metabolism of thyroid hormones, contributing to maintaining normal thyroid function (88). Thyroid dysfunction itself can adversely affect both metabolic profiles and ovulation in PCOS patients. Additionally, selenium and vitamin E show a well-established cooperative role in the antioxidant defense system. Vitamin E scavenges lipid peroxyl radicals within cell membranes, whereas selenium-dependent GPX subsequently suppress the resulting peroxides to harmless alcohols (24). Together, they constitute a critical cooperative antioxidant barrier for cellular protection.

Although selenium is an essential trace element, it possesses a relatively narrow therapeutic window. Chronic excessive intake, particularly when beyond 3 months or at daily doses exceeding 300 μg, increases the risk of selenosis (89), whose clinical manifestations may include abdominal symptoms such as vomiting, diarrhea, pain, and nausea as well as a garlic-like odor on the breath (90). Therefore, selenium supplementation in PCOS patients should adhere to recommended dosages, and prolonged unsupervised excessive intake should be avoided.

To conclude, selenium supplementation effectively counteracts metabolic disturbances integral to PCOS pathophysiology by reinforcing systemic antioxidant defenses and optimizing transcriptional regulation of glucose and lipid transporters.

### 5.2 Chromium

Chromium is an essential trace mineral found in broccoli, whole grains, meat, brewer’s yeast, and certain fruits. Nutritionally, the physiologically active form is trivalent chromium, a component of the glucose tolerance factor, which must be distinguished from the toxic industrial pollutant hexavalent chromium (91). Trivalent chromium plays an important role in glucose and lipid metabolism primarily by potentiating insulin signaling.

Clinical evidence supports the beneficial effects of trivalent chromium supplementation in ameliorating PCOS manifestations. A RCT demonstrated that daily supplementation with 200 μg of chromium for 8 weeks significantly improved fasting blood glucose (FBG), FINS levels, and HOMA-IR, increased the QUICKI in PCOS patients (92), while also showing a positive trend in regulating menstrual cyclicity and ovulation (93). Chromium supplementation significantly reduced serum levels of TG, VLDL, and TC, and was associated with a marked elevation in plasma TAC and a significant decrease in MDA levels (92). Another interventional study involving 35 adolescent PCOS subjects receiving 1,000 μg of chromium picolinate daily for 6 months reported significant reductions in ovarian volume, total follicle count, and FT levels, though no significant improvements were observed in acne or hirsutism scores (94).

The beneficial effects of chromium are mediated through multiple mechanisms. Its anti-inflammatory activity is reflected in the decrease of inflammatory markers, including hs-CRP, TNF-*α* and IL-6 levels, likely resulting from the suppression of inflammation-related mRNA expression and phosphorylation-dependent signal transduction (95). In terms of antioxidant capacity, chromium stimulates the activity of endogenous antioxidant enzymes, thereby raising TAC while diminishing harmful products such as MDA and ROS, collectively inhibiting lipid peroxidation (92, 96). At the signaling pathway level, chromium impedes kinase cascades that impair insulin signaling, consequently improving insulin sensitivity in peripheral tissues. These integrated mechanisms ultimately contribute to the amelioration of lipid metabolism and IR (96, 97).

Chromium interacts synergistically with several other nutrients. Vitamin C has been shown to promote the absorption of trivalent chromium and confer protective effects against hexavalent chromium-induced hepatotoxicity (98). In addition, adequate zinc intake alongside chromium supplementation plays a significant role in mitigating chromium-induced cytotoxicity, OS, and apoptosis (99).

Regarding potential risks, trivalent chromium nutritional supplements—particularly chromium picolinate—have a high safety profile within guideline-recommended doses. One report indicated that it is among the least toxic nutrients, with no significant toxic risk observed even at daily intakes of 1,000 μg (100). However, as a heavy metal, chronic exposure and bioaccumulation of chromium can lead to significant toxicity and diverse pathophysiological impairments, including allergic reactions, anemia, dermal burns, and ulcerative lesions—particularly in the stomach and small intestine—as well as spermatogenic damage and male reproductive system dysfunction, ultimately affecting multiple biological systems (101). Therefore, supplementation should be conducted under medical supervision, and toxic forms such as hexavalent chromium must be strictly avoided.

Collectively, supplementation with physiological doses of trivalent chromium may positively influence the metabolic and endocrine disturbances in PCOS through its insulin-sensitizing, anti-inflammatory, and antioxidant properties. However, the optimal dosage, long-term efficacy, and underlying molecular mechanisms require further validation through larger-scale, high-quality clinical investigations.

### 5.3 Zinc

Zinc, the second most abundant essential trace element in humans, is widely distributed in various food sources. It is particularly abundant in animal-based products such as oysters, red meat, and poultry, while legumes, nuts, and whole grains serve as excellent plant-derived sources. Functionally, zinc plays pleiotropic roles by binding to over 300 enzymes and more than 2,000 transcription factors (102). It is critically involved in a wide range of biological processes, including modulation of OS, maintenance of cellular homeostasis, immune regulation, DNA replication and damage repair, cell cycle progression, apoptosis, and aging (103).

A meta-analysis revealed that women with PCOS have significantly lower serum zinc levels compared to healthy controls (104). Clinically, zinc supplementation has been well-documented to confer metabolic benefits in PCOS patients. A RCT showed that daily supplementation with 220 mg zinc sulfate (equivalent to 50 mg elemental zinc) for 8 weeks led to marked improvements in multiple metabolic parameters relative to placebo. Specifically, PCOS women receiving zinc exhibited significant decreases in FBG, FINS, HOMA-IR, HOMA-*β*, TG, and VLDL, along with a significant increase in the QUICKI (105). In another randomized, double-blind, placebo-controlled trial, twice-daily supplementation with 250 mg of magnesium oxide plus 220 mg of zinc sulfate (equivalent to 50 mg elemental zinc) for 12 weeks significantly reduced serum hs-CRP and protein carbonyl (PCO) levels and markedly elevated plasma TAC compared with the placebo (106).

Zinc exerts its beneficial effects through multi-level mechanistic pathways. As an antioxidant, it acts as an essential cofactor for key antioxidant enzymes, orchestrating systemic redox homeostasis by modulating SOD activity, regulating the glutathione disulfide (GSSG)/GSH ratio, enhancing GPX and CAT activities, and inhibiting NADPH oxidase activation, thereby effectively reducing intracellular MDA and ROS levels (107, 108). In insulin signaling, zinc activates the insulin-dependent tyrosine kinase pathway and promotes the phosphorylation of PI3K and AKT, fine-tuning the insulin receptor signaling cascade to improve both insulin secretion and peripheral sensitivity (32). Regarding its anti-inflammatory properties, zinc participates in the regulation of inflammation-related genes and transporter proteins, suppresses enzymes involved in inflammatory signal transduction, and downregulates the NF-κB pathway, ultimately leading to attenuate secretion of pro-inflammatory cytokines (108, 109). Moreover, Zinc inhibits apoptosis and stimulates cell survival by modulating pro-survival pathways, stabilizing p53 function, maintaining B-cell leukemia/lymphoma 2 (BCL-2) family balance, and directly blocking caspase activity (110).

Zinc interacts closely with several other nutrients. A competitive antagonism exists between zinc and copper during intestinal absorption, whereby long-term, high-dose zinc supplementation may induce copper deficiency, potentially leading to adverse effects (111). Furthermore, zinc displays an antagonistic relationship with toxic heavy metals like cadmium; adequate zinc nutritional status helps reduce cadmium absorption and accumulation, and mitigate its toxicity (112).

The World Health Organization (WHO) recommends a daily dietary zinc intake of 6.7–15 mg. Regulatory bodies have established distinct tolerable upper intake levels, with the European Food Safety Authority (EFSA) setting a 25 mg/day and the U. S. Food and Drug Administration (FDA) permitting up to 40 mg/day. Although zinc is essential for physiological functions, prolonged excessive intake may impair immune response, disrupt the absorption of other trace elements—particularly copper—and increase the risk of adverse outcomes such as anemia, neutropenia, and copper deficiency (113). Therefore, zinc supplementation in PCOS patients should be administered within recommended dosage ranges under appropriate guidance, and long-term high-dose use should be avoided.

In conclusion, zinc supplementation can effectively ameliorate core pathological features of PCOS through multiple mechanisms, including the regulation of OS, attenuation of chronic inflammation, and optimization of insulin metabolic pathways. Given its well-defined mechanisms and favorable safety profile, zinc holds considerable promise for broader application in the clinical nutritional management of PCOS.

### 5.4 Calcium

Calcium is essential not only for bone development, coagulation, and cardiac function but also serves as a ubiquitous intracellular second messenger, playing a pivotal role in nerve impulse transmission, muscle contraction, secretory activities, cellular metabolism, immune responses, and enzyme activation (114). Dietary sources rich in calcium primarily include dairy products, dark green leafy vegetables, soy products, nuts, and fortified foods.

Emerging evidence points to a plausible link between dysregulated calcium metabolism and PCOS pathogenesis. Insufficient calcium availability may compromise the enzymatic conversion of androgens to estrogen within granulosa cells, consequently disrupting folliculogenesis and ovulation in affected individuals (115). Notably, epidemiological studies identify calcium deficiency as the most prevalent mineral inadequacy among women with PCOS, frequently concomitant with perturbations in vitamin D and PTH signaling (116).

Therapeutic intervention with calcium supplementation has demonstrated significant clinical benefits. In overweight/obese PCOS women with vitamin D deficiency, daily administration of 1,000 mg of calcium combined with 50,000 IU of vitamin D weekly for 8 weeks revealed that calcium supplementation, with or without additional vitamin D, resulted in significant diminutions in FBG, FINS, HOMA-IR, TG, and VLDL-C, along with a notable increase in the QUICKI (117). Another study reported that weekly 50,000 IU vitamin D₃ for 8 weeks, followed by daily oral supplementation with 2,500 mg calcium carbonate (1,000 mg elemental calcium) plus 9.68 mg cholecalciferol (880 IU vitamin D₃) for 3 months, significantly elevated SHBG levels and decreased TT, FT, and ADMA concentrations, effectively improving menstrual regularity and alleviating hirsutism in non-obese PCOS patients (116). Although these outcomes cannot be attributed solely to calcium, they underscore its contributory role in the synergistic regulation of reproductive and metabolic abnormalities in PCOS.

Mechanistically, calcium supplementation enhances tyrosine phosphorylation of IRS while inhibiting the activation of serine/threonine kinases, thereby maintaining the integrity of the PI3K/AKT/GLUT insulin signaling pathway and improving insulin sensitivity (118). Calcium supplementation also promotes calcium influx, activating the PKC pathway. This interacts with the cyclic adenosine monophosphate/protein kinase A (cAMP/PKA) pathway or, through APPL1-dependent signaling, stimulates the generation of IP₃ and DAG. These integrated signaling events amplify the ovarian responsiveness to FSH, contributing to the improvement of ovulatory function in PCOS (115, 119, 120). Parallelly, increased intracellular calcium concentration activates calcium/calmodulin-dependent protein kinase II (CaMKII), downregulating Caspase-3 expression and subsequently inhibiting apoptosis in ovarian granulosa cells (121).

Calcium metabolism and absorption depend on synergistic interactions with other nutrients. Vitamin D is a key regulator of calcium absorption, significantly enhancing its active transport in the intestine. Magnesium exhibits both synergistic and antagonistic relationships with calcium in terms of neuromuscular excitability and skeletal health. Importantly, an appropriate calcium-to-magnesium ratio of 2:1 is recommended, as magnesium deficiency may impair the physiological functions of calcium (122).

The tolerable upper intake level for calcium is approximately 2,500 mg/day for adults. Long-term excessive calcium supplementation, particularly without adequate concomitant vitamin K2 intake, may elevate the risk of vascular calcification. Calcium supplements are associated with a higher incidence of gastrointestinal-related adverse events, described as constipation, abdominal cramping, bloating, upper gastrointestinal events, gastrointestinal disorders, digestive symptoms, and in some cases, severe diarrhea or abdominal pain (123).

Collectively, calcium and vitamin D typically act synergistically to mitigate the reproductive and metabolic disturbances in PCOS by modulating sex hormone balance, improving insulin sensitivity, and optimizing lipid metabolism. In clinical practice, combining sufficient dietary calcium intake with adequate vitamin D status represents a crucial integrated nutritional strategy for the comprehensive management of PCOS.

### 5.5 Magnesium

Magnesium is the fourth most abundant cation in the human body and a major intracellular divalent cation. As an essential cofactor for more than 300 enzymes, it participates extensively in critical physiological processes, particularly in regulating metabolic and homeostatic balance across all tissues (124). Natural food sources rich in magnesium include dark green leafy vegetables (such as spinach), nuts, seeds, legumes, whole grains, and dark chocolate.

Studies have shown that serum magnesium concentrations are significantly lower in women with PCOS compared to healthy controls (125). Another study indicated that PCOS women intake less dietary fiber and magnesium than non-PCOS controls, and those with IR have even lower magnesium intake and a higher dietary glycemic load than their non-IR counterparts. Magnesium intake was also inversely correlated with IR, CRP, and T levels, and positively correlated with HDL-C (126), suggesting that increased magnesium intake may help ameliorate IR, hyperandrogenemia, and dyslipidemia in PCOS.

Recent meta-analyses have yielded divergent conclusions regarding the efficacy of magnesium supplementation in PCOS. One revealed that magnesium combined with other nutrients significantly improves glycolipid metabolism (127), while another found no significant effect of magnesium supplementation alone on cardiovascular metabolic risk factors or hormonal parameters (128). These discrepancies may discrepancies may stem from the number of available studies, population heterogeneity, and variations in dosage and intervention duration. However, clinical intervention studies have confirmed the benefits of magnesium supplementation. A RCT demonstrated that daily supplementation with 250 mg /day of magnesium oxide for 2 months effectively reduced FINS, HOMA-IR, TC, LDL, and FBG concentrations, while simultaneously increasing HDL levels in PCOS patients (129). Subsequent studies manifested that a 12-week intervention with a combination of magnesium/magnesium oxide (250 mg), vitamin E (400 mg), zinc (50 mg), and calcium (400 mg)-vitamin D (200 IU) collectively improved multiple parameters, including significantly decreased in hirsutism scores, hs-CRP, PCO, and MDA, along with simultaneously increased in plasma NO and TAC (106, 130, 131). Furthermore, it downregulated the gene expression of pro-inflammatory cytokines such as IL-1 and TNF-*α* (106), implying that magnesium not only independently regulates glucose and lipid metabolism but also displays synergistic effects with other nutrients to ameliorate inflammatory and OS.

While the precise molecular mechanisms are not fully elucidated, key pathways have been identified. Magnesium deficiency inhibits the conversion of vitamin B1 to thiamine diphosphate, diminishing pyruvate dehydrogenase activity. This disruption promotes excessive hepatic production of NADPH, subsequently stimulating the synthesis of TG and VLDL, and exacerbating lipid disorders (132). Analogously, magnesium is an essential cofactor for the autophosphorylation of insulin receptor tyrosine kinase—a process directly associated with enhanced insulin sensitivity, which further triggers the downstream PI3K/AKT signaling pathway and induces the translocation of GLUT4 to the cell membrane, enhancing cellular glucose uptake and regulating blood glucose levels (107).

Magnesium maintains close relationships with calcium and vitamin D. Adequate magnesium is critical for the activation of vitamin D in the hydroxylation processes, while both nutrients jointly regulate calcium metabolism and bone health (133). High-dose zinc supplementation may competitively inhibit intestinal magnesium absorption, highlighting the importance of maintaining a balanced intake of these minerals.

Dietary magnesium intake is generally considered very safe. The 2015–2020 Dietary Guidelines for Americans establish the recommended magnesium intake for adult women at 320 mg/day (134). However, when consumed in supplemental form, doses exceeding 350 mg/day in adults may cause gastrointestinal adverse effects such as diarrhea and nonspecific mild abdominal pain (135). Patients with severe renal impairment require extreme caution due to the risk of magnesium accumulation and toxicity.

To sum up, magnesium supplementation illustrates clear potential in alleviating PCOS symptoms by modulating glucose and lipid metabolism, improving insulin signaling transduction, and exerting synergistic anti-inflammatory and antioxidant effects. Therefore, conducting more high-quality clinical trials to precisely evaluate its efficacy in PCOS management and further elucidating its underlying molecular mechanisms are of significant importance for clarifying its clinical application value.

### 5.6 Iron

Iron is a crucial nutrient and a component of ferroproteins and enzymes that carry out vital biochemical functions (136). Its dietary sources include heme iron—found in red meat, animal offal, and blood-based products, which exhibits higher bioavailability—and non-heme iron (from plant sources such as spinach, legumes, and fortified grains).

A meta-analysis revealed that serum iron and ferritin concentrations are significantly elevated in women with PCOS compared to healthy controls (137). Another study reported a positive correlation between serum ferritin and the severity of menstrual dysfunction, indicating that iron retention resulting from chronic oligomenorrhea may contribute to increased iron storage in some PCOS patients (138). The underlying mechanisms for iron overload are multifaceted: firstly, chronic oligomenorrhea reduces physiological iron loss, causing acquired iron accumulation; secondly, decreased circulating levels of hemoglobin-binding proteins such as haptoglobin (HP) and α2-macroglobulin (A2M) lead to abnormally increased intestinal iron absorption via the free hemoglobin pathway; thirdly, dysregulation of hepcidin disrupts iron recycling within macrophages, consequently boosting plasma iron concentrations (139). Moreover, studies have found that compared to healthy controls, PCOS patients exhibit downregulated expression of genes including ferritin heavy chain 1 (FTH1), HAMP, GPX4, A2M, and HP, and upregulated expression of nuclear receptor coactivator 4 (NCOA4), along with enhancement in lipid peroxidation, serum ferritin, and total protein content, and inhibition in peroxidase activity (140).

Research denotes that compensatory hyperinsulinemia in PCOS promotes intestinal iron absorption, while iron overload, in turn, exacerbates IR and metabolic abnormalities, thereby disrupting insulin signaling and creating a vicious cycle (141, 142). In parallel, iron overload can trigger ferroptosis, an iron-dependent form of programmed cell death. In the ovary, ferroptosis induces mitochondrial dysfunction and excessive ROS production, impairing the activity of respiratory chain complexes and key enzymes, leading to cellular damage, exacerbated OS, and adverse effects on follicular growth, oocyte maturation and quality, fertilization, and embryonic development (143).

In clinical practice, given the prevalent trend of iron overload in PCOS patients, routine iron supplementation is generally not recommended unless iron deficiency anemia is confirmed (144). For patients with confirmed iron overload accompanied by clinical symptoms, therapeutic phlebotomy or dietary interventions to reduce iron intake under medical supervision may be exploratory strategies to improve metabolic and ovulatory function.

Iron interacts significantly with several other nutrients in the diet. Vitamin C potently stimulates the absorption of non-heme iron, while divalent cations such as calcium and zinc may compete for intestinal absorption pathways. High intake of dietary fiber, phytates, and polyphenols—commonly present in tea and coffee—can also inhibit iron absorption (145). The primary concern for the PCOS population is that indiscriminate iron supplementation may induce or exacerbate iron overload, amplifying its pro-OS effects.

In conclusion, a complex and bidirectional relationship exists between iron dysregulation—particularly iron overload—and the metabolic abnormalities and reproductive impairments in PCOS. However, the specific regulatory network of iron metabolism in PCOS has not yet been fully elucidated, and studies evaluating the potential benefits of reducing iron load, whether using animal models or PCOS populations, remain relatively scarce. Therefore, the role of iron homeostasis in PCOS pathogenesis and its potential as a therapeutic target undoubtedly represent an important direction for future investigation.

## 6 Bioactive compounds and PCOS

### 6.1 Melatonin

Melatonin, a neuroendocrine hormone characterized by its circadian rhythmicity and potent endogenous antioxidant activity, is primarily secreted by the pineal gland. Besides endogenous synthesis, it is also present in various foods, including meats, fish, eggs, cereals, tubers, oilseeds, mushrooms, fruits, vegetables, and dairy products (146). Research has demonstrated that melatonin concentrations in the follicular fluid of PCOS patients are significantly lower than in healthy women, and its levels correlate positively with basal FSH, suggesting a potential involvement of melatonin deficiency in the reproductive pathology of PCOS (147).

Clinical intervention studies have preliminarily revealed the potential therapeutic potential of melatonin in PCOS. A meta-analysis confirmed that melatonin supplementation significantly increases TAC in PCOS patients (148). A clinical study observed that PCOS patients receiving 5 mg of melatonin twice daily for 12 weeks showed significant decreases in hirsutism, serum TT, hs-CRP, and plasma MDA levels, along with increased plasma TAC and total GSH levels. Melatonin supplementation also downregulated gene expression of IL-1 and TNF-*α* compared to placebo (149). Another study indicated that combined supplementation of melatonin (6 mg) with magnesium oxide (250 mg) for 8 weeks produced more pronounced enhancements in TAC and greater improvement in hirsutism, while serum TNF-α levels were reduced with melatonin either alone or in combination with magnesium (150). These findings collectively hint that melatonin may alleviate clinical manifestations of PCOS through its anti-inflammatory and antioxidant pharmacological activities.

Melatonin exerts its beneficial effects on PCOS via multiple interconnected pathways. In ameliorating IR, it activates the PI3K/AKT signaling cascade, upregulating the mRNA and protein expression of IRS-1 and GLUT4 while downregulating phosphorylated IRS-1 (p-IRS-1 at Ser307), thereby augmenting cellular glucose uptake (151). Regarding gut microbiota modulation, melatonin stimulates the relative abundance of beneficial bacteria such as *Lachnospiraceae* and *Ruminococcaceae* while attenuating potentially harmful taxa including *Prevotellaceae*, *Muribaculaceae* and *Leuconostocaceae*. These changes are associated with regulated tryptophan metabolism and steroid hormone synthesis, resulting in improved endocrine function (152). In ovarian protection, melatonin promotes the phosphorylation of malic enzyme 1 (Mel), modulates NADPH levels, and enhances the activity of antioxidant enzymes—including SOD, CAT, GPX and glutathione reductase (GR)—along with GSH content., collectively restoring mitochondrial function and lessening granulosa cell apoptosis (152). Additionally, melatonin suppresses excessive autophagy through upregulation of the PI3K/AKT pathway (153).

Melatonin exhibits potential synergistic effects with several other nutrients. As previously noted, it demonstrates a synergistic relationship with magnesium in boosting antioxidant defenses (150). Parallelly, folic acid and vitamin B6 serve as pivotal cofactors for synthesis of serotonin from tryptophan. Zinc and magnesium, in turn, are considered to promote the conversion of serotonin to melatonin by binding to and activating arylalkylamine N-acetyltransferase (AANAT) (154). Thus, adequate levels of these vitamins and minerals are crucial for maintaining physiological melatonin concentrations.

Regarding safety, short-term use (less than 1 month) of melatonin at recommended doses (typically 0.15 mg to 12 mg daily) is generally well-tolerated. Commonly reported adverse effects include daytime drowsiness, headache, dizziness, hypothermia, and other sleep-related events, which are usually mild and self-limiting (155). However, safety data on long-term, high-dose melatonin use remain limited, particularly in women of reproductive age. Therefore, supplementation should be supervised medically, with attention to its potential effects on circadian rhythms.

In brief, melatonin supplementation may effectively ameliorate multiple pathological processes of PCOS through various pathways, including anti-inflammatory and antioxidant effects, regulation of glucose metabolism, and modulation of gut microbiota. However, current evidence is largely limited to animal models or small-scale clinical observations. Future large-scale, multicenter RCTs are urgently needed to robustly evaluate the efficacy and safety of melatonin in treating PCOS and to further elucidate its mechanisms across diverse ethnic populations, thereby establishing a more comprehensive evidence base for its potential clinical application.

### 6.2 *ω*-3 polyunsaturated fatty acids

ω-3 PUFAs are a class of unsaturated fatty acids with significant physiological importance, which primarily include alpha-linolenic acid (ALA), derived mainly from plant oils, as well as eicosapentaenoic acid (EPA) and docosahexaenoic acid (DHA), obtained predominantly from marine fish, algae, and krill oil (156). Due to their notable anti-inflammatory and antioxidant properties, *ω*-3 PUFAs have attracted considerable research interest for their potential role in ameliorating PCOS.

A Mendelian randomization analysis demonstrated that genetically predicted higher levels of *ω*-3 PUFAs are associated with a significantly reduced risk of developing PCOS (157). Observational studies investigating the relationship between ω-3 PUFAs and PCOS have found that ω-3 PUFAs intake is inversely correlated with HOMA-IR, fat mass, and body fat percentage. Higher levels of both EPA and DHA were individually associated with lowered HOMA-IR, while dietary and serum *ω*-3 PUFAs showed a positive correlation with muscle mass and negative correlations with fat mass and body fat percentage (158). In clinical intervention, daily supplementation with 2.4 g of EPA + DHA (at a ratio of 1.49:1) for 6 weeks resulted in a significant decrease in plasma bioavailable testosterone in PCOS patients (159). Multiple meta-analyses revealed that *ω*-3 PUFAs supplementation significantly lessened the HOMA-IR, improved lipid profile by lowering TC and TG, and diminished androgen levels (TT and DHEA) (160, 161). Additional meta-analysis reported that ω-3 PUFAs supplementation decreased serum levels of CRP, while increasing the secretion of the anti-inflammatory adipokine (adiponectin) in women with PCOS (162), and significantly improved pregnancy rates and conception rates among women undergoing fertility treatments (163).

The mechanisms of ω-3 PUFAs in ameliorating PCOS involve multiple signaling pathways and physiological processes. Firstly, ALA mitigates LPS-induced damage through dual anti-inflammatory and antioxidant mechanisms. Its anti-inflammatory effect is mediated by suppressing the TLR4/MyD88/NF-κB pathway, which inhibits the phosphorylation of downstream p65 and inhibitor of NF-κB (IκBα), causing downregulated MCP-1 expression and upregulated IL-13 secretion. This is complemented by its antioxidant activity, characterized by reduced ROS generation, enhanced SOD activity, and decreased MDA accumulation (164). In steroidogenesis regulation, ALA downregulates the transcription of key genes such as *CYP11A1*, reducing excessive steroid hormone production and subsequently improving endocrine disturbances in PCOS (165). Secondly, in improving insulin sensitivity and lipid metabolism, EPA and DHA activate the G protein-coupled receptor 120 (GPR120) and PPAR-*γ* pathways, upregulating the expression of insulin signaling molecules and promoting the browning of white adipose tissue, thereby ameliorating IR and adipose tissue inflammation (166). Combined application of DHA and EPA synergistically activates the IRS-1/PI3K pathway, stimulating downstream expression of GLUT4 and adiponectin, leading to comprehensive improvement of IR and dyslipidemia (167). Finally, in gut microbiota modulation, *ω*-3 PUFAs increase the abundance of beneficial bacteria such as *Bifidobacterium* and *Lactobacillus*, and inhibit the growth of potentially harmful bacteria like *Escherichia coli* and *Enterococcus*, subsequently reducing the release of pro-inflammatory cytokines including TNF-*α*, CRP, and IL-6, and improving systemic immune and inflammatory (168).

*ω*-3 PUFAs present a synergistic interaction with other nutrients, particularly vitamin D. As an anti-inflammatory and antioxidant agent, vitamin D is often co-administered with *ω*-3 PUFAs to prevent their auto-oxidation—a vulnerability conferred by multiple double bonds—preserving their structural integrity and bioactivity, and exhibiting complementary anti-inflammatory and apoptotic regulatory effects (169). *ω*-3 PUFAs can be co-administered with vitamin E, underscoring superior efficacy in improving hormonal profiles and glucolipid metabolism compared to monotherapy (22).

ω-3 PUFAs supplementation is generally safe at recommended doses. Commonly reported adverse effects are mild, including diarrhea and taste disturbances. High-dose ω-3 PUFAs may exert an antiplatelet aggregation effect, increasing bleeding tendency. Thus, high-dose supplementation should be administered under medical supervision with appropriate monitoring of coagulation parameters (170).

ω-3 polyunsaturated fatty acids suggest clear potential in ameliorating metabolic and endocrine disturbances in PCOS through their multi-faceted mechanisms, including anti-inflammatory and antioxidant effects, modulation of insulin signaling, improvement of lipid metabolism, and remodeling of gut microbiota. However, current studies are generally limited by relatively small sample sizes and short follow-up durations. Therefore, future large-scale, multicenter RCTs with extended follow-up periods are urgently needed to rigorously evaluate the long-term efficacy, safety, and durability of *ω*-3 PUFAs in the PCOS population, thereby providing more robust evidence for their clinical application.

### 6.3 Coenzyme Q10

Coenzyme Q10 (CoQ10), an endogenous fat-soluble quinone localized in the mitochondrial inner membrane, is a key cofactor in cellular energy metabolism and ATP synthesis. Dietary sources include animal offal (such as heart and liver), deep-sea fish, meat, spinach, and broccoli (171). CoQ10 supplementation has been associated with a reduction in all-cause mortality events (172), and its antioxidant properties contribute to improvements in cardiovascular diseases and inflammatory outcomes (173), hinting at therapeutic potential for MetS, diabetes, and human fertility disorders.

Clinical studies have confirmed the therapeutic value of CoQ10 in PCOS. A RCT in overweight/obese PCOS patients found that 200 mg/day CoQ10 supplementation for 8 weeks significantly reduced serum levels of inflammatory markers, including TNF-*α*, hs-CRP, and IL-6, along with markers of vascular endothelial dysfunction such as intercellular adhesion molecule-1 (ICAM-1) and E-selectin (174). Another RCT reported that 100 mg/day of CoQ10 for 12 weeks decreased hs-CRP, TT, dehydroepiandrosterone sulfate (DHEAS), hirsutism scores, and MDA, while simultaneously increasing SHBG and TAC compared to placebo (175). A meta-analysis concluded that CoQ10 supplementation effectively improves IR parameters (reducing HOMA-IR, FINS, and FBG), regulates sex hormone profiles (elevating FSH and decreasing T), and optimizes lipid metabolism (lowering TG, TC, and LDL while raising HDL levels) in women with PCOS (176).

Mechanistically, in antioxidant activity, as a potent antioxidant, CoQ10 activates the Kelch-like ECH-associated protein 1 (KEAP1)/Nrf2/ antioxidant response element (ARE) signaling pathway, upregulating the expression and enhancing the activity of downstream antioxidant enzymes such as SOD, NQO1, and HO-1. This cascade effectively scavenges ROS and ameliorates the systemic OS status characteristic of PCOS (177). Regarding metabolic regulation, it improves insulin sensitivity by modulating insulin signaling pathways (involving elements such as IRS and GLUT2) and lessening adipokine secretion (178). Concurrently, it activates AMPK and PPAR-*α*, leading to the downregulation of sterol regulatory element-binding protein 1c (SREBP-1c) expression (179). In anti-inflammatory, it also inhibits the activation of transcription factor activator protein-1 (AP-1) and the NF-κB signaling pathway, promoting fatty acid oxidation, suppressing lipid peroxidation and synthesis, and alleviating cellular inflammation (180).

CoQ10 exhibits noteworthy interactions with other nutrients. Its endogenous biosynthesis requires the participation of multiple vitamins as essential cofactors. Furthermore, as a fat-soluble compound, concurrent supplementation with vitamin E has been shown to produce more pronounced improvements in SHBG levels in PCOS patients compared to CoQ10 monotherapy (181). This combination also manifest synergistic efficacy in attenuating the HOMA-IR, implying complementary mechanisms in addressing key metabolic and endocrine disturbances in PCOS (182).

CoQ10 has a favorable safety and tolerability profile at recommended dosages (typically around 100 mg/day), with studies indicating no significant adverse effects even following prolonged exposure to doses as high as 900 mg/day (171).

Although CoeQ10 has been established as a nutrient possessing multifaceted physiological properties—including antioxidant, immunomodulatory, anti-inflammatory, and lipid-regulating activities—the comprehensive mechanistic network underlying its beneficial effects in PCOS remains incompletely elucidated. Future well-designed fundamental investigations and clinical trials are still warranted to thoroughly decipher the potential biological links between CoQ10 and PCOS pathophysiology, and to define its optimal interventional strategies.

### 6.4 N-acetyl cysteine

N-Acetylcysteine (NAC) is a synthetic compound and an acetylated derivative of the natural amino acid L-cysteine. As a classic mucolytic agent, NAC has long been used clinically for the treatment of acetaminophen intoxication and respiratory conditions (183). The core of its biological activity lies in its role as a precursor to GSH, the primary intracellular antioxidant. By providing the cysteine necessary for GSH synthesis, NAC directly scavenges ROS and exerts broad antioxidant and anti-inflammatory effects via the modulation of redox-sensitive signaling pathways (183).

Study has revealed that GSH levels in both serum and follicular fluid are significantly lower in PCOS patients versus healthy controls, accompanied by elevated OS markers such as MDA and decreased paraoxonase-1 activity, indicating a decline in the body’s antioxidant defense capacity and an exacerbation of lipid peroxidation (184). This reflects the depletion of the GSH defense system and is closely associated with core pathophysiological processes, including IR, chronic inflammation, and impaired follicular development in PCOS.

Multiple studies have confirmed the beneficial effects of NAC supplementation in PCOS. In a letrozole-induced PCOS mouse model, 160 mg/kg/day NAC for 12 days significantly ameliorated estrous cycle irregularities and ovarian morphology, reduced serum concentrations of LH, the LH/FSH ratio, and T, improved glucose clearance and insulin sensitivity, decreased ROS levels in oocytes and enhanced mitochondrial membrane potential (185). In the same study, PCOS women receiving 1.8 g/day NAC during ovulation induction required less mean FSH dosage and had shorter treatment duration, with significantly improved clinical pregnancy rates and the cumulative clinical pregnancy rates (185). A meta-analysis showed that NAC significantly lowered TT and increased FSH levels (186). Another meta-analysis found that NAC was associated with a statistically significant elevation in P and endometrial thickness in PCOS patients versus placebo and other drugs (metformin and clomiphene) (187). Compared to metformin, NAC resulted in significantly greater diminutions in BMI, body weight, FINS, the FBG/FINS ratio, TC, TG, and LDL. NAC also significantly lowered FBG levels compared to either metformin or placebo. However, NAC reduced HDL levels in PCOS women relative to metformin (188).

The mechanisms by which NAC ameliorates PCOS involve multiple interconnected pathways. In enhancing antioxidant defense, NAC serves as a direct precursor for GSH synthesis. By elevating intracellular GSH levels, NAC directly neutralizes excess ROS, mitigating oxidative damage to follicular development and insulin signaling pathways. NAC also activates the Nrf2 signaling pathway, upregulating the expression of various endogenous antioxidant enzymes and establishing long-term cytoprotection (189). Regarding the improvement of insulin sensitivity, OS is a key driver of IR. By scavenging ROS, NAC suppresses the activation of stress kinases, preserving the phosphorylation status of serine/threonine sites on IRS and promoting normal insulin signal transduction, eventually enhancing glucose uptake and utilization in peripheral tissues (190). From the perspective of anti-inflammatory effects and hormonal regulation, NAC inhibits the activation of the NF-κB signaling pathway, reducing the production of downstream pro-inflammatory cytokines including TNF-*α* and IL-6, and alleviating the chronic low-grade inflammation associated with PCOS (183).

As a broad-spectrum antioxidant, NAC may act synergistically with other antioxidant nutrients, potentially enhancing the body’s total antioxidant capacity through complementary mechanisms. Selenium serves as an essential component of GPX, and when combined with vitamin E, their combined antioxidant effect substantially surpasses that of vitamin E alone. Given that NAC functions as a direct precursor for GSH synthesis, its interaction with these nutrients may more effectively counteract the OS characteristic of PCOS (191). This synergistic antioxidant interplay underscores the potential advantage of multi-nutrient intervention strategies over single-agent supplementation in addressing the complex redox imbalance in this condition.

NAC has a favorable safety profile at recommended doses (typically not exceeding 1800 mg daily), maintaining good tolerability even at 3 g/day. The exceptionally low incidence of reported adverse events—with only four cases of heartburn documented at this higher dosage—collectively indicates excellent tolerability of NAC supplementation (192). However, due to its characteristic thiol group, NAC may possess an unpleasant sulfur-like odor. In rare instances, high-dose intravenous administration has been associated with hypersensitivity reactions, though these are uncommon with oral formulations. Notably, as NAC may potentiate the effects of certain hypoglycemic or antihypertensive medications, close monitoring of blood glucose and blood pressure is advisable during co-administration. Consultation with a healthcare professional is recommended prior to its long-term use in PCOS management.

Current evidence robustly denotes that NAC, as a multifaceted antioxidant agent, exerts comprehensive therapeutic benefits in PCOS via improving insulin sensitivity, attenuating OS and inflammation, and restoring sex hormone balance. However, its optimal dosage, long-term efficacy, and impact on critical endpoints such as live birth rates require further validation through larger, rigorously designed clinical trials.

## 7 Conclusion

**Figure 1:**
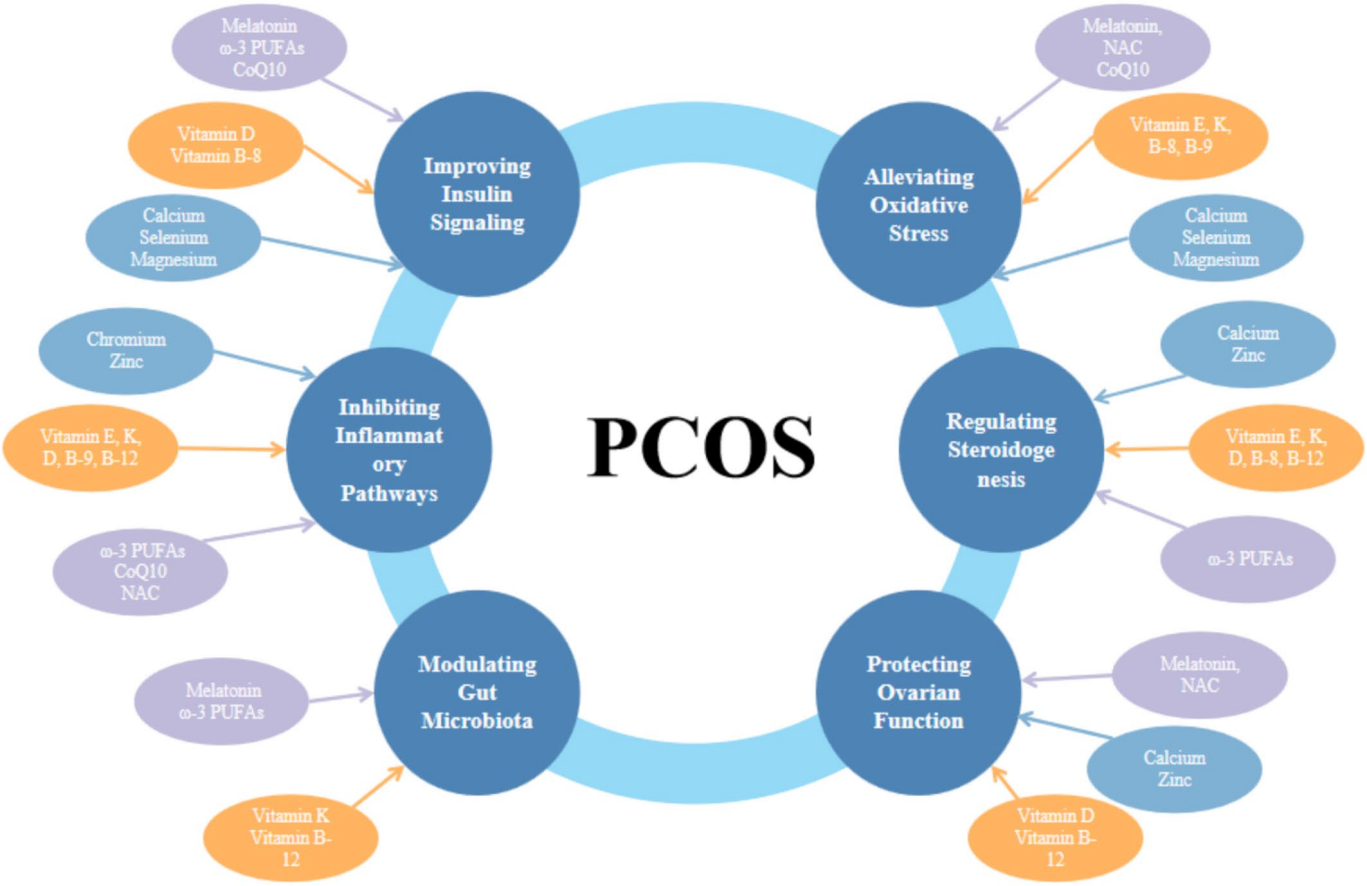
Nutritional and bioactive compound interventions on core pathophysiological mechanisms of PCOS.

PCOS represents a pervasive and complex endocrinopathy affecting reproductive-aged women worldwide. Its intrinsic pathophysiological heterogeneity—manifested through divergent metabolic, reproductive, and inflammatory phenotypes—continues to challenge the development of universally effective therapeutic strategies. Emerging evidence posits that targeted nutritional interventions—encompassing fat-soluble vitamins (D, E, K), water-soluble vitamins (B8, B9, B12), essential minerals (zinc, selenium, chromium, calcium, magnesium), and bioactive compounds (melatonin, *ω*-3 PUFAs, CoQ10, NAC)—may mitigate core PCOS manifestations including IR, dyslipidemia, HA, and chronic inflammation (Table 1). The pleiotropic benefits of these nutrients are achieved through the modulation of a network of critical signaling pathways, including insulin signaling, antioxidant defense, inflammatory cascades, and hormonal regulation, as schematically illustrated in Figure 1.

**Table 1:** Regulation of nutrients and bioactive compounds in PCOS.

Nutrients	Pathways	Indicators	Reference
Vitamin E	——	TG↓, VLDL-C↓, TC↓, LDL↓, TC/HDL-C ratio TAC↑, MDA↓	16–27
	——	PPAR-γ↑, IL-8↓, TNF-α↓	
	——	T↓, LH↓, LDL↓, TG↓, WC↓, HOMA-IR↓ P↑, FSH↑	
	——	TG↓, LDL↓, TC↓, hs-CRP↓, hirsutism scores↓ NO↑	
Vitamin K	——	FINS↓, HOMA-IR↓, HOMA-β↓, TG↓, DHT↓, FAI↓, body fat mass↓ QUICKI↑, SHBG↑, skeletal muscle↑	28–39
	NLRP3/ NF-κB	IL-6↓, TNF-α↓	
	Antioxidant Defense	SOD↑, GSH↑, GPX↑, CAT↑, iNOS↓, COX-2↓, p38 MAPK↓, ROS↓, caspase-1↓	
	Gut Microbiota	*Ruminococcaceae*↑, *Lactobacillaceae*↑ *Desulfovibrio*↓, *Escherichia coli*↓	
Vitamin D	——	25-(OH) D3↑, LH/FSH ratio↓, LH↓, T↓ pregnancy rates↑	40–51
	——	BMI↓, waist-to-hip ratio↓, FINS↓, HOMA-IR↓, TG↓, TC↓, LDL-C↓	
	——	endometrial thickness hs-CRP↓, PTH↓, TC↓, TT↓	
	RAGE	AGEs receptor↓, androgen↓ PI3K↓, AKT↓	
	MAPK	PGC-1α↑, NRF-1↑, ROS↓	
Inositol (Vitamin B-8)	——	BMI↓, HOMA-IR↓, insulin↓, TT↓, FT↓, LH↓, SHBG↑	52–63
	——	androstenedione↓, blood glucose↓	
	——	AMH↓, HOMA-IR↓	
	——	clinical pregnancy rates↑, high-quality embryo rates↑ antral follicles↓, AMH↓	
		GLUT4↑, IP₃↑, DAG↑, PKC↑, FSH↑, androgen↓,	
	FOXO/DAF-16 Nrf2/SKN-1	SOD↑, CAT↑, ROS↓, MDA↓	
	——	ATP↑, GSH↑, ROS↓	
	——	TNF-α↓, IL-6↓	
Folic Acid (Vitamin B-9)	——	hs-CRP↓, IL-18↓, TNF-α↓, insulin↓, HOMA-IR↓, TC↓, LDL↓ Hcy↓	65–70
	NF-κB	ROS↓, Hcy↓ TNF-α↓, IL-6↓, IL-1β↓	
	AMPK	ACC↑, CPT1↑	
	Nrf2	HO-1↑, NQO1↑, MDA↓	
Vitamin B-12	——	MMA↓, CPT1↑, fatty acid β-oxidation↑, IR↓	72–79
	——	AMH↓, 17-hydroxyprogesterone↓	
		CRP↓, TNF-α↓, IL-6↓	
	Firmicutes-to-Bacteroidetes ratio	*Faecalibacterium*↑, *Bacteroides*↓, SCFAs↑	
Selenium	——	TAC↑, TT↓, cholesterol↓	24, 80–91
	——	ADMA↓, TT↓, ApoB/ApoA1 ratio↓	
	——	PPAR-γ↑, GLUT1↑,	
	——	LDL receptor↓, AKT↓, AMPK↓	
	——	GPX1↑, H₂O₂↓	
Chromium	——	FBG↓, FINS↓, HOMA-IR↓ QUICKI↑	92–102
	——	TG↓, VLDL↓, TC↓ TAC↑, MDA↓	
	——	ovarian volume↓, total follicle count↓, FT↓	
	——	hs-CRP↓, TNF-α↓, IL-6↓	
	——	TAC↑, MDA↓, ROS↓	
Zinc	——	FBG↓, FINS↓, HOMA-IR↓, HOMA-β↓, TG↓, VLDL↓ QUICKI↑	32, 103–114
	——	hs-CRP↓, PCO↓, TAC↑	
	——	SOD, GSSG/GSH ratio, GPX↑, CAT↑ NADPH oxidase, MDA↓, ROS↓	
	——	SOD↑, CAT↑, GST↑, HOMA-IR↓, FINS↓, TG↓	
	——	PI3K↑, AKT↑	
	——	NF-κB↓	
	——	P53↓, BCL-2↓, caspases↓	
Calcium	——	FBG↓, FINS↓, HOMA-IR↓, TG↓, VLDL↓ QUICKI↑	115–124
	——	SHBG↑, TT↓, FT↓, ADMA↓	
	IRS	PI3K↑, AKT↑, GLUT↑	
	cAMP/PKA	PKC↑, IP₃↑, DAG↑, FSH↑	
	Calcium/CaMKII	Caspase-3↓, apoptosis↓	
Magnesium	——	FINS↓, HOMA-IR↓, TC↓, LDL↓, FBG↓, HDL↑	107, 108, 125–136
	——	hirsutism scores↓, hs-CRP↓, PCO↓, MDA↓ NO↑, TAC↑	
	——	IL-1↓, TNF-α↓	
	——	NADPH↓, TG↓, VLDL↓	
	PI3K/AKT	GLUT4↑	
Iron	Free hemoglobin	HP↓, A2M ↓	137–146
	——	hepcidin↓	
	Iron metabolism related genes	FTH1↓, HAMP↓, GPX4↓, A2M↓, HP↓, NCOA4↑, ferritin↑, total protein↑, peroxidase↓	
	Ferroptosis	ROS↑	
Melatonin	——	TAC↑	147–156
	——	hirsutism↓, TT↓, hs-CRP↓, MDA↓, TAC↑, GSH↑ IL-1↓, TNF-α↓	
	——	TAC↑, hirsutism↓, TNF-α↓,	
	PI3K/AKT	IRS-1↑, GLUT4↑	
	Gut microbiota	*Lachnospiraceae*↑, *Ruminococcaceae*↑ *Prevotellaceae*↓, *Muribaculaceae*↓, *Leuconostocaceae*↓	
	——	Me1↑, NADPH↓, SOD↑, CAT↑, GPX↑, GR↑, GSH↑	
	PI3K/AKT	autophagy↓	
ω-3 polyunsaturated fatty acids	——	HOMA-IR↓, fat mass↓, body fat percentage↓, fat mass↓ muscle mass↑	157–172
	——	Bioavailable T↓	
	——	HOMA-IR↓, TC↓, TG↓, LDL↓, TT↓, DHEA↓	
	——	CRP↓, adiponectin↑	
	TLR4/MyD88/NF-κB	p65 ↓, IκBα↓, MCP-1↓, IL-13↑ ROS↓, SOD↑, MDA↓	
	——	CYP11A1↓	
	——	GPR120↑, PPAR-γ↑	
	IRS-1/PI3K	GLUT4↑, adiponectin↑	
	Gut microbiota	*Bifidobacterium*↑, *Lactobacillus*↑ *Escherichia coli*↓, *Enterococcus*↓	
Coenzyme Q10	——	TNF-α↓, IL-6↓, hs-CRP↓, ICAM-1↓, E-selectin↓	173–183
	——	hs-CRP↓, TT↓, DHEAS↓, hirsutism scores↓, MDA↓ SHBG↑, TAC↑	
	——	HOMA-IR↓, FINS↓, FBG↓, FSH↑, T↓ TG↓, TC↓, LDL↓, HDL↑	
	KEAP1/Nrf2/ ARE	SOD↑, NQO1↑, HO-1↑ ROS↓	
	——	IRS↑, GLUT2↑	
	——	AMPK↑, PPAR-α↑, SREBP-1c↓	
	——	AP-1↓, NF-κB↓	
N-Acetylcysteine	——	LH↓, LH/FSH ratio↓, T↓, ROS↓	185–195
	——	mean FSH dosage↓, clinical pregnancy rates↑, cumulative clinical pregnancy rates↑	
	——	TT↓, FSH↑	
	——	P↑, endometrial thickness↑	
	——	BMI↓, body weight↓, FINS↓, FBG/FINS ratio↓, TC↓, TG↓, LDL↓, FBG↓, HDL↓	
	Nrf2	GSH↑, ROS↓	
	——	IRS↑	
	NF-κB	TNF-α↓, IL-6↓	

“↑” = Upregulation, “↓” = Downregulation.

Nevertheless, the current data remains fragmented by considerable methodological heterogeneity. Variations in cohort characteristics—including age, BMI, and ethnic background—inconsistent application of diagnostic criteria, and unstandardized intervention protocols such as dose-duration ratios, combination therapies significantly limit the comparability of findings and obscure true effect sizes in existing meta-analyses.

To transcend these limitations, future research must adopt a multidimensional integrative framework to systematically enhance scientific rigor and translational value. This necessitates constructing precisely defined cohorts through phenotype-stratified enrollment combined with genomic stratification strategies, enabling targeted analysis of distinct PCOS subtypes. Concurrently, standardizing the assessment of metabolic assessments is critical: the hyperinsulinemic-euglycemic clamp should be universally prioritized as the gold standard for evaluating insulin sensitivity to minimize bias arising from methodological heterogeneity. Longitudinal studies integrating multi-omics approaches—including metabolomics and proteomics—are essential to systematically delineate the dynamic relationships between nutrient interventions and biomarker profiles and to elucidate the pharmacodynamic characteristics of these nutrients. Furthermore, mechanistic investigations should focus on: (1) elucidating the epigenetic regulation of steroidogenic enzymes by specific nutrients; (2) defining the role of mitochondrial metabolic reprogramming mediated by compounds like CoQ10 and essential minerals; and (3) unraveling the crosstalk within the gut-brain-ovary axis modulated by *ω*-3 PUFAs and other nutrients.

Ultimately, resolving these knowledge gaps will facilitate a transition toward a theranostic paradigm, wherein micronutrient supplementation is tailored to individual’s pathophenotype and underlying metabolic dysregulation. This evolution from empirical adjuncts to mechanism-based nutritional interventions holds significant promise for improving the precision and efficacy of PCOS management.
